# The Role of PAS Kinase in PASsing the Glucose Signal

**DOI:** 10.3390/s100605668

**Published:** 2010-06-04

**Authors:** Julianne H. Grose, Jared Rutter

**Affiliations:** 1 Department of Microbiology and Molecular Biology, Brigham Young University, Provo, UT 84602, USA; 2 Department of Biochemistry, University of Utah School of Medicine, Salt Lake City, UT 84112, USA; E-Mail: rutter@biochem.utah.edu

**Keywords:** PAS kinase, PASKIN, glucose sensor, protein phosphorylation, PAS domain, metabolic syndrome

## Abstract

PAS kinase is an evolutionarily conserved nutrient responsive protein kinase that regulates glucose homeostasis. Mammalian PAS kinase is activated by glucose in pancreatic beta cells, and knockout mice are protected from obesity, liver triglyceride accumulation, and insulin resistance when fed a high-fat diet. Yeast PAS kinase is regulated by both carbon source and cell integrity stress and stimulates the partitioning of glucose toward structural carbohydrate biosynthesis. In our current model for PAS kinase regulation, a small molecule metabolite binds the sensory PAS domain and activates the enzyme. Although bona fide PAS kinase substrates are scarce, *in vitro* substrate searches provide putative targets for exploration.

## 1. Introduction

### 1.1. Times of Plenty and Times of Scarcity

Glucose is an important source of energy and metabolic fuel in both prokaryotes and eukaryotes. When glucose enters the cell, it can be used in a variety of different processes. For example, the central metabolic pathways conserved throughout all domains of life, namely glycolysis, the citric acid cycle (TCA), and the pentose phosphate pathway, center around glucose consumption (catabolism) for the production of usable energy (ATP) and reducing equivalents (NAD(P)H). Glucose is also used in anabolic reactions such as the essential modification of proteins and the production of lipids and glycans for cellular proliferation. In addition to catabolic and anabolic reactions, glucose may be stored in carbohydrate form as glycogen or be converted to lipid for storage. Each of these processes must be regulated appropriately and dynamically to ensure maximum growth and survival of an organism through times of plenty and times of scarcity. This regulation often involves nutrient responsive protein kinases, which sense the cellular levels of metabolic intermediates and regulate metabolic pathways through protein phosphorylation.

Although the presence of glucose in the environment signals nutrient richness to most organisms and cell types, their responses to glucose can be dramatically different. For example, in most eukaryotic organisms, high glucose levels stimulate glucose oxidation along with storage in the form of glycogen or fat, using times of plenty to yield high levels of ATP as well as to store for “lean times”. In contrast, when glucose levels are high the single celled fungi *Saccharomyces cerevisiae* primarily ferments glucose and leaves glucose storage for leaner times [1]. Although far less energy (ATP) is produced from fermentative metabolism, the high flux through glycolysis may provide metabolic building blocks for rapid growth, allowing the yeast to outcompete other organisms through glucose depletion. Such fermentative growth is seen in many highly proliferative cancer cells and is known as the Warburg effect [2].

The differential response to glucose by different cell types requires glucose sensing mechanisms coupled with diverse modes of metabolic regulation. For example, the 5′-AMP Activated Protein Kinase (AMPK) responds to low glucose levels (via a high AMP: ATP ratio) and down-regulates ATP utilization pathways while simultaneously up-regulating ATP production pathways (for recent reviews see [3,4]). The mammalian Target of Rapamycin (mTOR) protein responds to a variety of inputs to up-regulate protein synthesis and cell growth and proliferation when nutrients abound [5,6]. This review focuses on PAS kinase, a nutrient sensing protein kinase that is involved in glucose homeostasis in yeast [7–10] and mammals [11–16].

### 1.2. PAS Kinase Structure

PAS kinase is broadly evolutionarily conserved amongst eukaryotes, having homologs in yeast, drosophila, mice and man, but is not found in *C. elegans*. Its sequence contains a C-terminal serine-threonine kinase domain and an N-terminal Per-ARNT-Sim (PAS) domain. By primary amino acid sequence, the serine-threonine kinase domain lies near the CAMK branch on the human kinome dendrogram [17]. The N-terminal PAS domain belongs to a large superfamily of PAS domains, comprising over 21,000 PFAM entries from all kingdoms of life [18,19]. PAS domains are sensory domains that frequently regulate an attached functional domain *in cis*, often by serving as a protein interaction surface. PAS domains are found attached to a variety of functional domains, including transcriptional activators, guanylate cyclases, phosphodiesterases, ion channels and kinases. Some PAS domains bind ligands within their cores allowing them to sense a variety of transient cellular and environmental conditions. PAS domains can either bind ligands reversibly like the citrate sensor CitA [20], or constitutively like the covalent binding of 4-hydroxycinnamic acid by the blue light sensing photoactive yellow protein [21,22] or the non-covalent heme-binding oxygen-sensing protein FixL [23]. PAS domains display low sequence conservation and high functional diversity, yet they contain a structurally conserved core of a five-stranded anti-parallel beta sheet surrounded by several alpha helices [19]. The conservation of general structure combined with the malleability of function make PAS domains good targets for structure-based design of artificial sensors where sensory PAS domains are covalently linked to effector domains of choice [24–26]. For example, Möglich *et al.* recently replaced the oxygen-sensing PAS domain of *Bradyrhizobium japonicum* FixL with the LOV photosensory PAS domain of *Bacillus subtilis*, resulting in a chimeric kinase that was regulated by blue light instead of oxygen [24]. In addition, a plant derived blue-light sensing PAS domain has been fused to dihydrofolate reductase (DHFR) from *E. coli*, a protein that is not normally regulated by PAS domains. Remarkably, this fusion protein exhibited DHFR activity that was modestly regulated by blue light even without optimization of the construct [25].

A high resolution NMR structure of the human PAS kinase (hPASK) PAS domain was solved in the lab of Dr. Kevin Gardner [27] and it was found to be similar to other PAS domains including the well-characterized FixL heme-based oxygen sensor of *Rhizobia* [27]. The hPASK PAS domain adopts the typical α/β PAS domain fold with several α helices (Cα, Dα, Eα and Fα) surrounded by a 5-stranded (Aβ, Bβ, Gβ, Hβ, and Iβ) antiparallel beta sheet. In addition, the PAS domain contains an unusually long and dynamic loop segment (Fα/FG loop). The structure of this region is unlike any other PAS domain for which a three-dimensional structure is available [27–29]. Interestingly, Gardner’s group demonstrated that the PAS domain binds the kinase domain, even when supplied *in trans*, primarily using residues within the FG loop [27]. The PAS domain had been previously shown to inhibit the kinase domain when added *in trans*, and the combined data suggested an inhibitory direct protein-protein interaction [15].

In addition to solving the apo PAS domain structure, the Gardner lab also identified specific PAS domain ligands from a small molecule library and determined the region of the protein involved in ligand binding [27]. The PAS domain from PAS kinase exhibited binding selectivity, even discriminating amongst structurally similar ligands [27]. Although the small molecule ligands identified are nonphysiological, these ligands were bound in a similar manner to the FixL and Phy3 ligands. Interestingly, ligand binding induces changes in the dynamics of the adjacent PAS domain FG loop and the surrounding region. In the FixL/heme complex, conformational changes in the FG loop, brought about by oxygen binding to the distal face of the heme, are thought to modulate the activity of the kinase domain [30,31]. A similar mechanism of kinase activity modulation is likely for PAS kinase as well since the FG loop was identified as the primary surface for kinase domain interaction. The structural details of how FG loop conformation regulates PAS kinase activity, however, might be quite distinct from FixL and other sensory histidine kinases, wherein effector domains are connected to the C-terminus of the PAS domain by short α-helical and coiled-coil linkers that are believed to transmit the PAS domain signal [19]. In contrast, PAS kinase has a long linker (at least 400 amino acids) and appears to display a direct interaction between the PAS domain and the effector (kinase) domain. Additional evidence for PAS domain regulation of kinase activity arises from the finding that PAS domain mutants, which were designed to mimic the ligand bound state (I203F and C228F), lead to increased PASK activity in the context of the full length protein or failed to inhibit kinase activity when supplied *in trans* [15,27].

### 1.3. A Model for PAS Kinase Activation and Function

**Figure 1.**
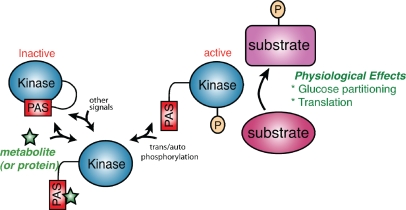
A model for PAS kinase regulation and function. The PAS domain binds to and inhibits the kinase domain. A metabolite (green star) or protein activates PAS kinase by binding to the PAS domain and relieving PAS domain inhibition. This transient activation may be subsequently stabilized through auto- or transphosphorylation. PAS kinase is then competent to phosphorylate substrates involved in glucose partitioning and translation (in *S. cerevisiae*) to elicit the appropriate physiological response.

In the current model of PAS kinase activation and function, the PAS domain binds to and inhibits the kinase domain (Figure 1). This binding and inhibition may be disrupted by the association of a small molecule metabolite or protein with the PAS domain. There is no evidence for a constitutively associated ligand from the biochemical and structural work and ligand binding might be transient as attempts at biochemical purification of a PAS domain ligand have failed (our unpublished data). Stable activation may, therefore, also entail subsequent auto- or transphosphorylation, as typical of many protein kinases. Human PAS kinase has been shown to autophosphorylate at multiple sites *in vitro* [15], and another phosphosite (Ser116) was detected in a large scale search for nuclear phosphoproteins in HeLa cells [32]. The functional importance of these phosphosites in the regulation of endogenous PAS kinase, however, remains unclear.

### 1.4. Expression and Subcellular Localization

**Figure 2.**
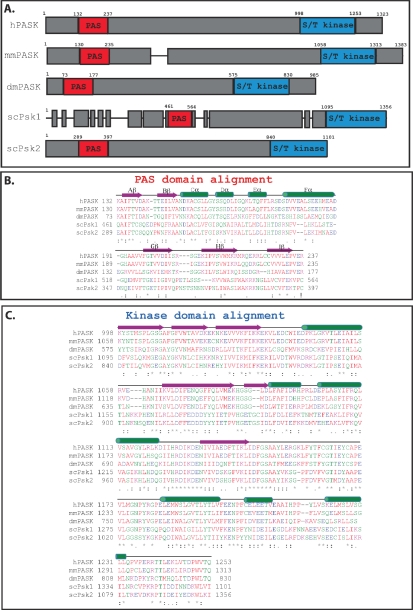
Alignment of the PAS and kinase domains from selected PAS kinase orthologs. (A) Schematic of PAS kinase homologs. Regions of similarity are boxed in grey, with non-homologous regions indicated by gaps between the grey boxes. Alignment of the PAS (B) and kinase (C) domains from selected PAS kinase orthologs. Secondary structure elements (α-helical and β-sheet) were derived from the published NMR structure of the PAS domain [27] as well as amino acid alignment of the kinase domain with the published structure of microtubule-associate protein/microtubule affinity regulating kinase 2 (MARK2) [33], and are shown above the amino acids in green and purple respectively. Amino acid sequences were aligned using the Clustal W program [34]. The degree of amino acid conservation is indicated both by color and “*” (identical residues in all sequences), “:” (highly conserved amino acids), and “.” (weakly conserved amino acids). Red indicates a small hydrophobic or aromatic amino acid (-Y), blue indicates acidic, magenta is basic, and green is hydroxyl plus amine plus basic (-Q), all other amino acids are grey.

The regions of PAS kinase with the greatest interspecies conservation lie within the PAS and kinase domains (Figure 2). For example the *S. cerevisiae* Psk1 and Psk2 kinase domains have 61% and 62% similarity to the human PASK kinase domain, respectively. The region between the PAS and kinase domains may be important for substrate recognition since it was shown to be critical for interaction between PAS kinase and one of its substrates [16]. PAS kinase is predominantly found in the cytoplasm of both HeLa Cells [15,35] and yeast cells [36], but was detected by mass spectrometry in HeLa cell nuclear extracts [32,35]. Studies in mice suggest that PAS kinase is expressed ubiquitously at a low level in tissues other than the testes, where the mRNA and protein level is roughly 100-fold higher than that of most other tissues [37,38].

## 2. PAS Kinase Glucose Sensing

### 2.1. Mammalian PAS Kinase

PAS kinase is rapidly (within 1 hour) post-translationally activated by high glucose in cultured mouse pancreatic beta-cells [11,12]. This activation is followed by an increase in both PAS kinase mRNA and protein (24 hours) and is independent of insulin concentration. The primary function of beta-cells is to synthesize and secrete insulin in response to high glucose levels. Through binding to its receptor on target cells, insulin stimulates the uptake of serum glucose by the muscle and adipose tissue and inhibits hepatic glucose production. In addition to causing insulin secretion, high glucose also stimulates transcription of the preproinsulin gene. PAS kinase overexpression mimics this effect of high glucose and causes induction of the preproinsulin promoter even in low glucose, while RNAi-mediated depletion of PAS kinase suppresses glucose-induced preproinsulin up-regulation [11].

Fatty acids impair the glucose responsiveness of insulin gene transcription. This and related phenomena, known as glucolipotoxicity, might underlie the pancreatic beta-cell failure observed in Type 2 Diabetes [39]. Similarly, the glucose-induced increase in PAS kinase gene expression was recently shown to be inhibited by the fatty acid palmitate [12]. Interestingly, PAS kinase overexpression mitigates the effects of palmitate on glucose-induced expression of preproinsulin and pancreatic duodenal homeobox-1 (PDX-1), a transcription factor required for preproinsulin expression and pancreatic beta cell function [12]. These observations raise the possibility that PAS kinase is a key mediator of the effects of glucose and fatty acids on insulin expression in pancreatic beta-cells. While the physiological significance is unclear, purified PAS kinase efficiently phosphorylates purified PDX-1 on Thr152 [40].

In addition to a role in beta-cell insulin gene expression, PAS kinase appears to function in peripheral tissues in regulating glucose homeostasis in mice. In light of the evolutionary conservation of PAS kinase, it was surprising that PASK knockout mice have no overt phenotype when fed a normal chow diet [13,41]. However, when these mice were challenged with a high-fat diet, they were protected from an array of phenotypes similar to the metabolic syndrome, including obesity, liver triglyceride accumulation, glucose intolerance and insulin resistance [13]. The most striking phenotype, protection from liver triglyceride accumulation, was accompanied by a significant decrease in the levels of mRNAs encoding SCD1, which catalyzes a rate limiting step in triglyceride biosynthesis, CD36, a putative fatty acid transporter, and PPARγ, which controls expression of the CD36 gene and other genes involved in fat accumulation [13]. Although the molecular mechanisms of these phenotypes are unknown, a higher metabolic rate may be involved since the PAS kinase-deficient mice display increased O_2_ consumption and CO_2_ release [13]. This higher metabolic rate is not due to an increase in mitochondria mass or number, or activation of the AMPK pathway in muscle or liver [13].

### 2.2 Yeast PAS Kinase

Yeast PAS kinase also appears to be regulated in response to high glucose, although in the opposite manner. The genome of the yeast *S. cerevisiae* encodes two homologs of PAS kinase, Psk1 and Psk2 (see Figure 2). Although highly similar in the PAS and kinase domains, Psk1 has an extra ∼250 amino acids distributed throughout the N-terminus. The yeast PAS kinase homologs are posttranslationally activated by two stimuli, growth on nonfermentative carbon source (carbon sources other than glucose) and growth under conditions of cell integrity stress [7]. When glucose is available, yeast preferentially ferment the glucose and repress the expression of over 100 genes involved in respiratory metabolism in a process known as glucose repression [1,42]. This process is under the control of the yeast AMPK homolog, Snf1, the master regulator of glucose repression. Snf1 is necessary and sufficient for PAS kinase activation by nonfermentative carbon sources; that is, PAS kinase is constitutively activated when Snf1 is constitutively activated (via a *reg1* mutation), while it is not activated in the *reg1 snf1* double mutant [7]. The activation of PAS kinase by nonfermentative growth conditions may be mechanistically related to the activation of PAS kinase by high glucose in mammalian beta-cells since both conditions activate respiratory metabolism.

Interestingly, only Psk1 is activated by nonfermentative carbon source due to down regulation of Psk2 transcription under these conditions, while both Psk1 and Psk2 are capable of responding to cell integrity stress [7]. The differential regulation of the two PAS kinase homologs argues for specialized functions, as does the evolutionary selection for both genes [8]. The presence of two PAS kinase homologs is most likely the result of a whole genome duplication that occurred in an *S. cerevisiae* ancestor [43]. Most of the genome duplication was subsequently deselected and lost by mutation, however, some of the redundant proteins evolved specialized functions that were selected and maintained [44–46]. Although both Psk1 and Psk2 phosphorylate Ugp1, the one definitively validated yeast substrate, specialized functions may be revealed as more substrates are identified.

## 3. PAS Kinase Regulation of Glucose Partitioning

### 3.1. Glucose Partitioning and the Regulation of Glycogen Biosynthesis

A biochemical screen of fractionated yeast proteins yielded four putative PAS kinase substrates: UDP-glucose pyrophosphorylase (Ugp1), eukaryotic translation initiation factor 1A (eIF1A or Tif11), cap-associated factor (Caf20), and Sro9, a ribosome-associated protein [9]. A summary of putative PAS kinase substrates is listed in Table 1. Of these four targets, only Ugp1 has been shown to be physiologically relevant as expression of the unphosphorylatable mutant (Ugp1-S11A) causes a phenotype identical to the *psk1 psk2* double PAS kinase mutant, namely sensitivity to cell wall perturbing agents and hyperaccumulation of glycogen [10]. Ugp1 is an essential protein that catalyzes the conversion of glucose-1-phosphate and UTP to UDP-glucose. Its product, UDP-glucose, is utilized as a glucose donor in a variety of cellular processes ranging from biosynthesis of glycogen and cell wall glycans to the glycosylation of proteins [47]. Surprisingly, PAS kinase-dependent phosphorylation of Ugp1 does not alter its enzymatic activity. Instead, phosphorylation alters the final destination of its product, favoring cell wall glucan biosynthesis at the expense of glycogen biosynthesis [10]. This altered glucose partitioning in response to phosphorylation most likely occurs through relocalization of cytoplasmic Upg1 to the cell periphery where it may interact with cell wall biosynthetic enzymes [10]. Thus, the observed activation of PAS kinase by growth conditions that elicit cell integrity stress (see above) would result in increased formation of cell wall glucans necessary for repair.

Glycogen is the predominant storage carbohydrate and, thus, the proper regulation of glycogen metabolism is essential to energy homeostasis in both yeast and mammals. Dysregulation of glycogen metabolism has been shown to play a role in the development of a variety of diseases, including type 2 diabetes [49–51]. While the Ugp1 Ser11 phosphorylation site is not found in mammals, the role of PAS kinase in the regulation of glycogen synthesis and glucose partitioning in general appears to be conserved. The enzyme glycogen synthase is the enzyme responsible for glycogen biosynthesis and is the key regulatory point for glycogen synthesis [52,53]. Glycogen synthase is regulated by multi-site phosphorylation in both yeast and mammals, with a total of nine phosphorylation sites identified. Glycogen synthase kinase-3 (GSK-3) has been shown to phosphorylate some of these sites, however, other glycogen synthase kinases must exist since glycogen synthase is phosphorylated at critical sites (Ser640 and Ser644) even when GSK-3 activity is blocked [54]. PAS kinase efficiently phosphorylates purified mammalian glycogen synthase at Ser640, the phosphosite with the most dramatic effects on activity and a PAS kinase/glycogen synthase interaction can be detected by copurification [54]. Interestingly, this phosphorylation requires a region upstream of the kinase catalytic domain (amino acids 444–955), as does copurification of PAS kinase with glycogen synthase [16]. This region appears to be responsible for the kinase/substrate interaction, which is disrupted by excess glycogen [16]. Yeast PAS kinase also phosphorylates glycogen synthase (Gsy2) *in vitro* and *psk1 psk2* mutant yeast display elevated glycogen synthase activity [9].

### 3.2. A Role for PAS Kinase in Translation

The three remaining putative yeast PAS kinase substrates identified in the biochemical screen were proteins involved in translation: eukaryotic translation initiation factor 1A (eIF1A or Tif11), cap-association factor (Caf20), and Sro9 [9]. The eIF1A protein generates the 40S ribosomal preinitiation complex by catalyzing the transfer of Met-tRNA [55,56]. In addition, it has been shown to be involved in a wide variety of processes including RNA synthesis, apoptosis, cytoskeletal organization, activation of the heat shock transcription factor, and proteasomal degradation of damaged proteins [57]. Human eIF1A also appears to be a PAS kinase substrate since it is phosphorylated *in vitro* (at Thr432) and interacts with PAS kinase in vivo as shown by both yeast two-hybrid and GST pull-down assays [35]. In addition, eIF1A colocalizes with PAS kinase at the mid-tail region of mature sperm, which contains cytoskeletal components as well as mitochondria and glycolytic enzymes. Interestingly, in contrast to glycogen synthase, eIF1A copurification occurred with either the PAS domain or the kinase domain, but not the intervening region of PASK [35].

The remaining two *in vitro* yeast substrates, Caf20 and Sro9, are also involved in translation. Caf20 is a phosphoprotein that negatively regulates cap-dependent translation by binding eIF4E and inhibiting formation of the eIF4E/eIF4G complex [58,59]. Subsequent experiments revealed that the Caf20 protein is not phosphorylated *in vitro* in the absence of its partner eukaryotic initiation factor 4E (eIF4E). Sro9 is a cytoplasmic protein of unknown function that associates with translating ribosomes [60]. It regulates Hap1 as a component of the HMC complex [61] and appears to be involved in actin filament organization [62].

An analogous *in vitro* screen using human PAS kinase and fractionated HeLa cell extracts also identified putative PAS kinase substrates that are involved in translation [48]. Human PAS kinase was shown to phosphorylate ribosomal proteins S2, S6, S8, S10, S14, S3A (RPS3A), basic transcription factor 3 (BTF3), and alanyl-tRNA synthetase (AlaRS) (see Table 1) [48]. Like the yeast Caf20, BTF3 contains a sequence that binds to eIF4E [63]. The eIF4E binding proteins generally regulate translation in response to cellular conditions and some tether eIF4E to specific mRNAs (for a recent review see [64]). The ribosomal protein S3A is also involved in translational initiation and binds the small ribosomal subunit and iMET [65,66]. In addition, AlaRS may play a more direct role in translational regulation since various tRNAs have also been shown to bind to specific mRNAs and inhibit their translation [67]. PAS kinase phosphorylates AlaRS on Ser732, which lies between the tRNA editing and oligomerization domains [48]. The physiological consequences of these translation-related phosphorylation events in both yeast and mammals are unknown. *In vivo* phosphorylation by PAS kinase may not regulate translation initiation or elongation rates. Probst *et al.* recently pointed out [48] that other reported ribosomal phosphorylation events do not change these reaction rates but instead alter the specificity of the ribosome for particular mRNAs [68].

Although further studies are necessary to elucidate the role of PAS kinase in translation, PAS kinase overexpression in yeast rescues the temperature sensitivity and protein synthesis defects of a strain lacking Stm1 (Tif3), the yeast eIF4B translation initiation factor [9]. Additionally, a high copy suppressor screen for genes that suppress the *psk1 psk2* double mutant phenotype when overexpressed yielded genes encoding proteins involved in translation initiation [9]. Many of the proteins discussed above also play a role in mRNA stability, which is closely linked to ribosomal function. Interestingly, glyceraldehyde-3-phosphate dehydrogenase (GAPDH) was also identified in the *in vitro* screen for PAS kinase substrates and has also been shown to play a role in mRNA stability by associating with polysomes and cis acting elements [69]. The GAPDH phosphorylation site (Thr237) is conserved across all species and positioned at the homotetramer interface.

## 4. Conclusions

The presence of both a sensory PAS domain and a canonical serine/threonine kinase domain posit a role for PAS kinase in metabolic regulation (see figure 1 for a summary of PAS kinase regulation and function). The regulation of PAS kinase in response to cellular status has been documented in both mammalian beta-cells and yeast, with the unifying theme of activation under conditions that induce respiration (high glucose in pancreatic beta cells and nonfermentative carbon sources in yeast). However, the precise role of PAS kinase as a metabolic sensor awaits the identification of a biological ligand (either a small molecule or protein) that binds to the PAS domain and activates the enzyme. This identification is a formidable task since the PAS domain/ligand interaction is most likely transient, as no protein or small molecule ligands have been discovered despite repeated attempts at biochemical purification (unpublished). Additionally, PAS kinase activation may require auto- or transphosphorylation and key PAS kinase residues have been shown to be phosphorylated, however the *in vivo* relevance of phosphorylation remains unknown [15,32]. The presence of a regulating ligand does not preclude phosphorylation as a mechanism to stabilize the activated state.

PAS kinase inhibition may prove to be a valid therapeutic target in the defense against the metabolic syndrome since PAS kinase-deficient mice are protected against weight gain, liver triglyceride accumulation and insulin resistance when fed a high-fat diet [13]. Intriguingly, these mice display no phenotype when fed a normal chow diet. The molecular mechanisms behind these phenotypes, specifically the identification of PAS kinase substrates and interacting partners, will provide valuable insight into PAS kinase function.

PAS kinase regulates glucose homeostasis in yeast primarily through the phosphorylation of Ugp1 [9,10]. Ugp1 is the cellular source of UDP-glucose, an essential carrier of glucose. Phosphorylation of Ugp1 directs the flow of glucose towards the biosynthesis of structural carbohydrates at the expense of glycogen [7,8,10]. Although the phosphorylation site (Ser11) is not conserved in mammalian cells, both yeast and mammalian glycogen synthase proteins are putative PAS kinase substrates [9,16]. Many proteins and pathways are well conserved from yeast to man. Thus, the pathways regulated by PAS kinase in yeast may be similar to the mammalian pathways even if the actual phosphorylated substrates may vary. Although *bona fide* PAS kinase substrates are scarce, the recent identification of PAS kinase-activating stimuli [8,11] and *in vitro* yeast and mammalian PAS kinase substrates [9,48] should greatly aid in elucidating its regulation and function.

## Figures and Tables

**Table 1.** *In vitro* and *in vivo* substrates of PAS kinase.

**Putative substrate**	**Site(s)**	**Species**	**Evidence**	**Reference**
**Glucose-partitioning**				
Pancreatic duodenal homeobox 1 (PDX1)	Thr152	*H. sapiens*	*in vitro* phosphorylation	[40]
Glycogen synthase (Gsy)	Ser640	*H. sapiens*	*in vitro* phosphorylation; copurification	[16]
	Ser654	*S. cerevisiae*	*In vitro* phosphorylation;	[9]
UDP-glucose pyrophosphorylase (Ugp1)	Ser11	*S. cerevisiae*	*in vitro* & *in vivo* phosphorylation	[7,9,10]
**Translation-related**				
Translation initiation factor eIF1A	Ser125	*S. cerevisiae*	*in vitro* phosphorylation	[9]
	Thr432	*H. sapiens*	*in vitro* phosphorylation; coimmunoprecipitation; colocalization; yeast 2-hybrid	[35]
Caf20	Ser58 Ser59 c-term*	*S. cerevisiae*	*in vitro* phosphorylation	[9]
Sro9	Thr101 Thr103	*S. cerevisiae*	*in vitro* phosphorylation	[9]
Alanyl tRNA Synthetase (Alars)	Ser732	*H. sapiens*	*in vitro* phosphorylation	[48]
Basic Transcription Factor 3(BTF3)	Thr38	*H. sapiens*	*in vitro* phosphorylation	[48]
Ribosomal Protein S3A (RPS3A)	Ser154	*H. sapiens*	*in vitro* phosphorylation	[48]
Ribosomal Protein S2		*H. sapiens*	*in vitro* phosphorylation	[48]
Ribosomal Protein S6		*H. sapiens*	*in vitro* phosphorylation	[48]
Ribosomal Protein S8		*H. sapiens*	*in vitro* phosphorylation	[48]
Ribosomal Protein S10		*H. sapiens*	*in vitro* phosphorylation	[48]
Ribosomal Protein S14		*H. sapiens*	*in vitro* phosphorylation	[48]

* multiple phosphorylation events on the C-terminal 68 amino acids were reported.
